# 

**Published:** 1857-08

**Authors:** 


*Davis' Theory of the Circulation* is almost as good as Mrs. Willard's. For the benefit of the ignorant among our readers, we give the essence of it.

"Life is a substance, although, not unlike matter, it obeys a *higher law* than gravitation. * * * * The heart throws the blood to the finest ramifications of the vascular system, and magnetically calls it back again. * * * * This organ, unlike a *force pump*, operates upon positives and negatives, by alternate contractions and expansions. * * * * The visible heart performs this function because there is a corresponding spiritual heart within it," &c., &c.

Clear as mud. A chimera ruminating in a vacuum devours second intentions, we have heard, but how he does it is just as plain as Davis' theory of the circulation.

---

*Hard Times.*—Have our debtors forgotten us? We sent out bills to the amount of $2500, with the May number, but have not received enough to pay the running expenses since. This is rather hard upon a poor man. Perhaps we can hold out faithful to the end, living on air, but should any one see our obituary they may rest assured we died of starvation. Oh, for double our present number of advance paying subscribers. They are men after our own heart.

